# Predicting body weight of Kalahari Red goats from linear body measurements using data mining algorithms

**DOI:** 10.14202/vetworld.2022.1719-1726

**Published:** 2022-07-21

**Authors:** Kwena Mokoena, Kagisho Madikadike Molabe, Mmakosha Cynthia Sekgota, Thobela Louis Tyasi

**Affiliations:** Department of Agricultural Economics and Animal Production, University of Limpopo, Private Bag X1106, Sovenga 0727, South Africa

**Keywords:** body length, data mining algorithms, heart girth, rump height, withers height

## Abstract

**Background and Aim:**

The Kalahari Red goat breed is the finest meat-producing species in South Africa, and its coat color ranges from light to dark red-brown. A practical approach to estimating their body weight (BW) using linear body measurements is still scarce. Therefore, this study aimed to determine the best data mining technique among classification and regression trees (CART), Chi-square automatic interaction detection (CHAID), and exhaustive CHAID (Ex-CHAID) for predicting the BW of Kalahari Red goats.

**Materials and Methods:**

This study included 50 Kalahari Red goats (does = 42 and bucks = 8) aged 3–5 years. Body length (BL), heart girth (HG), rump height (RH), height at withers (WH), sex, and age were the essential indicators to estimate BW. The best model was chosen based on the goodness of fit, such as adjusted coefficient of determination (Adj. R^2^), coefficient of determination (R^2^), root mean square error (RMSE), standard deviation ratio (SD ratio), mean absolute percentage error, Akaike information criteria, relative approximation error, and coefficient of variation.

**Results:**

The SD values of the ratio ranged from 0.32 (CART) to 0.40 (Ex-CHAID). The greatest R^2^ (%) was established for CART (89.23), followed by CHAID (81.99), and the lowest was established for Ex-CHAID (81.70). CART was established as the preferred algorithm with BL, HG, and WH as critical predictors. The heaviest BW (73.50 kg) was established in four goats with BL higher than 92.5 cm.

**Conclusion:**

This study reveals that CART is the optimum model with BL, HG, and WH as the essential linear body measurements for estimating BW for Kalahari Red goats. The updated records will assist the rural farmers in making precise judgments for various objectives, such as marketing, breeding, feeding, and veterinary services in remote areas where weighing scales are unavailable.

## Introduction

Goats are valuable for commercial and subsistence farming methods in South Africa [1]. They provide milk and meat; their by-products are used to produce belts, whereas their feces are used as fertilizer in some countries. In addition, goats provide income, poverty relief, and food security to rural families [2]. The Kalahari Red goat is a local and meat-type goat species that originated in South Africa [3]. According to Snyman [4], the Kalahari Red goat specie is the optimum meat-producing breed in South Africa, with a coat color ranging from light to dark red-brown. Furthermore, they are characterized by a long, deep body with a medium-to-large frame and sturdy legs that permit them to wander far to search for varied vegetation [5].

The body weight (BW) of livestock is one of the crucial and important tools that should be recognized when conducting activities such as administering medicine doses, adjusting feed supply, evaluating growth, and selecting replacements [6]. Linear body measurement growth is a good indicator of animal welfare and a predictor of BW and carcass characterized for the future [7]. However, due to scarce resources, such as weighing scales, farmers depend on age and inspection to pick which one to sell [8]. The correlation is a statistical method that only describes the association between two variables; however, it does not demonstrate the resultant impact relationship between the variables [9]. Therefore, statistical algorithms, such as classification and regression tree (CART), Chi-square automatic interaction detection (CHAID), and exhaustive CHAID (Ex-CHAID), were suggested. These statistical algorithms are a tree-based model which assesses characteristics (independent traits) and other factors, such as gender, contributing an essential role to the dependent variable [10]. The decision tree is extensively applied to estimate BW from categorical and numerical-dependent variables in Boer goats [11], Balochi sheep [12], Beetal goats of Pakistan [13], and Turkish Tazi dogs [14] to help farmers in remote areas who do not have weighing scales.

This study aimed to formulate a model for evaluating the BW from body length (BL), heart girth (HG), withers height (WH), head width (HW), and rump height (RH) of Kalahari Red goats using data mining algorithms. The results obtained from this research will evaluate the BW of the Kalahari Red goat and help mainly rural farmers make knowledgeable decisions regarding breeding, marketing, feeding, and treating their animals.

## Materials and Methods

### Ethical approval

The study was approved by the University of Limpopo Animal Research Ethics Committee project number AREC/14/2021: PG.

### Study period and area

The study was conducted in May 2021. The present study was performed at Zuurfontein farm in Polokwane. The temperature in winter ranges from 7°C to 21°C and in summer ranges from 16°C to 28.1°C and experiences an annual rainfall of more than 600 mm [15].

### Experimental animal

The research used 50 Kalahari Red goats (42 does and 8bucks) 3–5 years of age. Kalahari Red goat is an optimum breed because it consumes plants and grass and needs low management and care [16].

### Animal management

The animals were managed under an extensive farming system in which they were allowed to go out in the morning to graze and return to their camps in the afternoon. Goats were monitored twice a day, in the morning, before they left for grazing, and in the afternoon, when they returned to their camps. Clean water was provided *ad libitum*.

### Data collection

**Figure-1:**
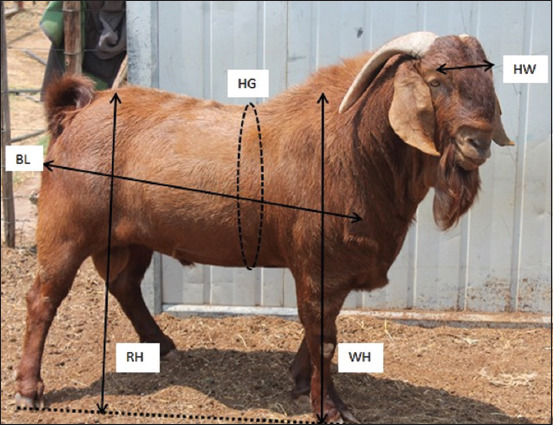
Kalahari Red goat showing linear body measurement traits measured.

The BW and linear body measurement properties of 50 Kalahari Red goats were estimated. The linear body measurement characteristics investigated are as follows: BL, HG, HW, RH, and WH, as shown in Figure-1. These measurements were conducted according to the suggestions of Tyasi *et al*. [17]. The linear body measurement dimensions were recorded in centimeters and evaluated using a flexible tape and wood ruler. In contrast, BW was described in kilograms using a weighing scale that does not impart pain to the animals. The data were extracted following the protocols of the Animal Ethics Committee.

### Statistical analysis

Statistical package for the social sciences v.27.0 (IBM Corp., NY, USA), with a probability of 5% for significance, was used to analyze the data. The decision tree algorithms were used to design the model for the predictive performance of BW using the study of Eyduran *et al*. [18]. The cross-validation was kept at 10, as recommended by Celik and Yilmaz [19]. The following goodness of fit was used to execute the predictive performance of CART, CHAID, and Ex-CHAID [10].

1. Coefficient of determination (R^2^):
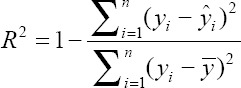
2. Adjusted coefficient of determination (Adj. R^2^):
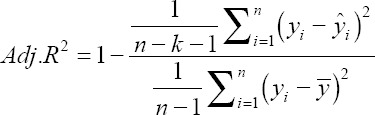
3. Root-mean-square error (RMSE):
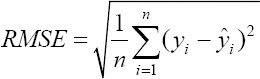
4. Standard deviation ratio (SD_ratio_):
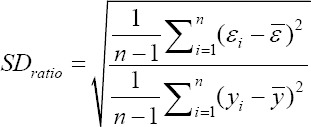
5. Mean absolute percentage error (MAPE):
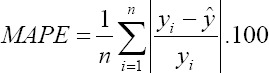
6. Akaike information criteria (AIC):
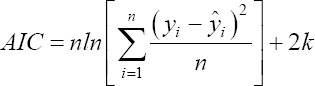
7. Relative approximation error (RAE):
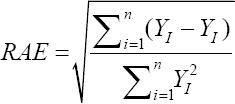
8. Coefficient of variation (CV):
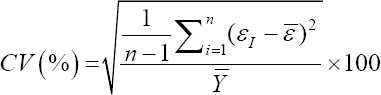

## Results

### Descriptive statistics of Bucks

Table-1 is a descriptive summary of the estimated variables of bucks. Buck average for BW was calculated to be 61.75 kg. HG had the greatest average of 99.00 cm, followed by BL with 88.50 cm and HW at 17.63 cm, which was calculated to have the least average value. The coefficient of variation of bucks ranged from 8.26% to 22.88%.

**Table 1:** Descriptive statistics of body weight and linear body measurement traits of bucks.

Traits	Mean	SD	CV (%)	Minimum	Maximum
BW (kg)	61.75	14.13	22.88	36.00	77.00
RH (cm)	76.75	6.34	8.26	68.00	87.00
HG (cm)	99.00	10.54	10.65	77.00	110.00
BL (cm)	88.50	10.27	11.60	76.00	100.00
HW (cm)	17.63	2.45	14.41	14.00	21.00
WH (cm)	74.38	8.31	11.17	64.00	86.00

BW=Body weight, RH=Rump height, HG=Heart girth, BL=Body length, HW=Head width, WH=Withers height, SD=Standard deviation, CV=Coefficient of variation

### Descriptive statistics of the Does

Table-2 summarizes the descriptive statistics of BW and linear body measurement properties of does. The mean value of the BW weight of the does was calculated to be 48.99 kg. HG had the maximum mean value of 84.69 cm, followed by BL, with an average value of 79.14 cm. Nevertheless, HW had the lowest mean value of 14.60 cm. The coefficient of variation ranges from 7.35% to 13.45%.

**Table 2:** Descriptive statistics of body weight and linear body measurement traits of does.

Traits	Mean	SD	CV	Minimum	Maximum
BW (kg)	48.99	6.59	13.45	38.00	60.00
RH (cm)	68.71	5.05	7.35	55.00	80.00
HG	84.69	7.26	8.57	71.00	105.00
BL	79.14	6.37	8.05	60.00	90.00
HW	14.60	1.12	7.67	13.00	18.00
WH	66.71	5.07	7.60	54.00	79.00

BW=Body weight, RH=Rump height, HG=Heart girth, BL=Body length, HW=Head width, WH=Withers height, SD=Standard deviation, CV=Coefficient of variation

### Correlation between BW and linear body measurement traits of does

**Figure-2:**
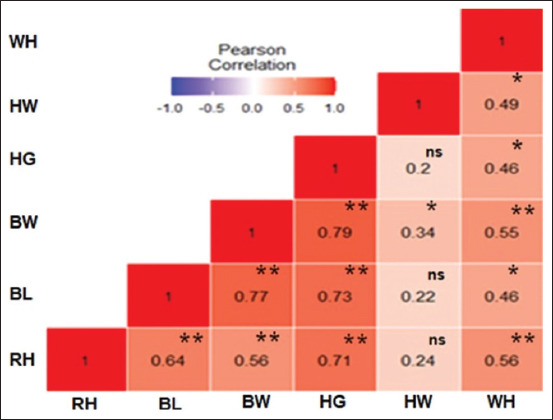
Heat map of Pearson’s correlation between body weight and measured variables of does. Heat map color distribution, high correlation is red color, mid-correlation is white color, and low correlation is blue color. BW=Body weight, RH=Rump height, HG=Heart girth, BL=Body length, HW=Head width, WH=Withers height, ^ns^Not significant, **Highly correlation, *Correlated.

The layout in Figure-2 establishes a link between the BW and linear body measurements. A positive statistical correlation was shown between BW and HW (p < 0.05). Nevertheless, the BW significantly correlated with HG, BL, RH, and WH (p < 0.01). The association between linear body measurements was discovered, where RH had a positively high statistical correlation with HG, BL, and WH (p < 0.01). Meanwhile, it showed no considerable correlation toward HW. BL was positively and statistically correlated with WH at p < 0.05, highly positively and statistically correlated with HG at p < 0.01 but demonstrated no considerable correlation toward HW. HG was positively correlated with WH at p < 0.05. However, it had no substantial association with HW; finally, HW was positively correlated with WH at p < 0.05.

### Correlation between BW and linear body measurement traits of bucks

**Figure-3:**
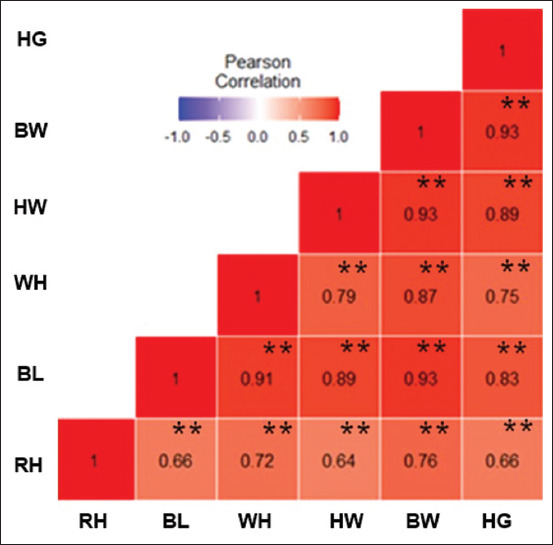
Heat map of Pearson’s correlation between body weight and linear body measurements traits of bucks. Heat map color distribution, high correlation is red color, mid correlation is white color, and low correlation is blue color. BW=Body weight, RH=Rump height, HG=Heart girth, BL=Body length, HW=Head width, WH=Withers height, ^ns^Not significant, **Highly correlation, *Correlated.

Figure-3 shows the association between BW and linear body measurement characteristics of bucks. The correlation results of Pearson depicted BW to have a highly positive statistical link with all linear body measurements at p < 0.01. All measured characteristics were highly and positively statistically correlated.

### Classification and regression tree model of the Kalahari Red goat

**Figure-4:**
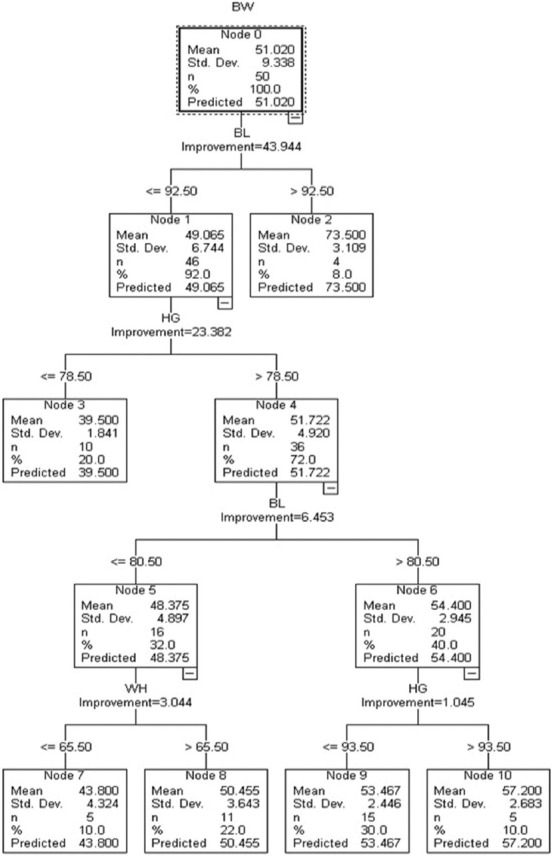
Classification and regression trees algorithm.

The CART model (Figure-4) of the Kalahari Red goat has BW as the dependent variable and body properties as independent properties. CAR trees comprise 10 nodes (nodes 2, 3, 7, 8, 9, and 10), being the terminal nodes. Node 0, as the root node, comprises the descriptive statistics of BW (mean = 51.03 kg, standard deviation = 9.34, n = 50 and estimated BW mean of 51.03). Node 0 was subdivided into node 1 (≤92.5 cm) and node 2 (>92.5 cm) through BL. Node 1 was subdivided on the bases of HG into node 3 (≤78.5 cm) and node 4 (>78.5 cm). Node 4 was subdivided into node 5 (≤80.5 cm) and node 6 (>80.5 cm) through BL. Node 5 was subdivided into node 7 (≤65.5 cm) and node 8 (>65.5 cm). Finally, node 6 was subdivided based on HG into node 9 (≤93.5 cm) and node 10 (>93.5 cm). Among terminal nodes, node 2 was the optimum node as it produced the maximum estimated mean BW (73.50 kg) than node 3 (39.50 kg), node 7 (43.80 kg), node 8 (50.45 kg), node 9 (53.50 kg), and node 10 (57.20 kg).

### CHAID and EX-CHAID models of Kalahari Red goats

**Figure-5:**
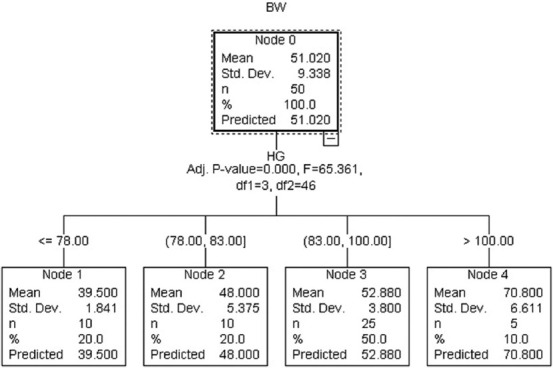
Exhaustive Chi-square automatic interaction detection algorithm.

**Figure-6:**
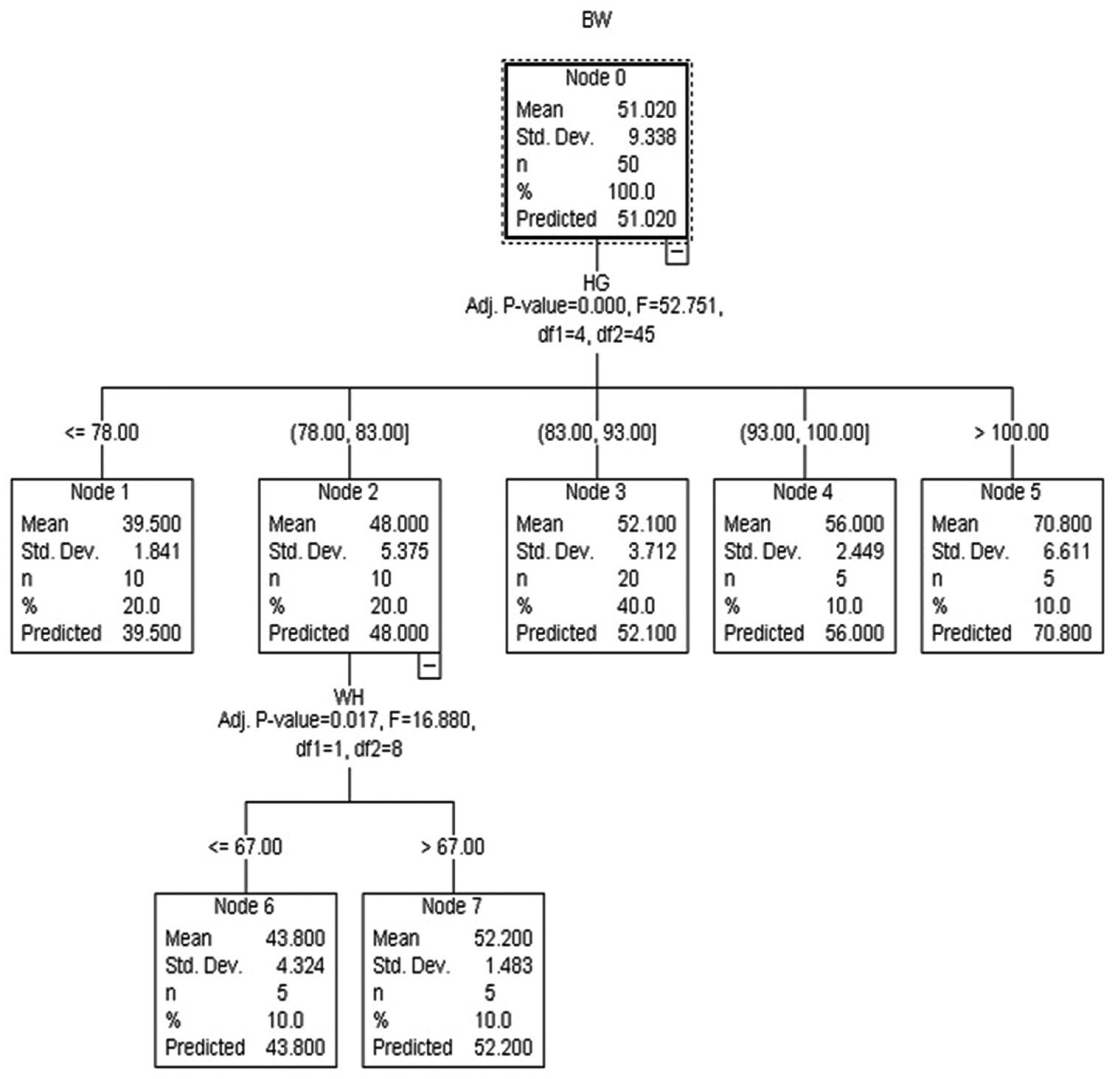
Chi-square automatic interaction detection algorithm.

The Ex-CHAID algorithm (Figure-5) comprised HG as a critical variable to estimate BW in Kalahari Red goats. The root node comprised descriptive statistics with HG subdivided the root node into nodes 1–4. Node 4 (>100 cm) estimated the highest BW with node 3 (83 cm < HG < 100 cm), node 2 (78 cm < HG < 83 cm), and node 1 (<78 cm), respectively. CHAID algorithm (Figure-6) contained HG and WH as substantial predictors of BW. The goats were subdivided into four nodes according to HG, and node 2 was further grouped into subgroups (nodes 6 and 7). Node 1 had HG <78 cm with an estimated BW of 39.5 kg, while node 2 and node 3 had 78 cm < HG < 83 and 83 cm < HG < 93 cm with BW of 48 kg and 52.13 kg, respectively. Node 4 had the maximum predicted BW of 70 kg with >100 cm HG. Nodes 6 and 7 were grouped according to WH with <67 cm WH that had 43.8 estimated BW and node 7 (>67 cm) with BW of 52.2 kg.

### Comparison of the CART, CHAID, and Ex-CHAID algorithms

Table-3 presents the goodness of fit criteria to evaluate the predictive performance between CART, CHAID, and Ex-CHAID. Among the statistical algorithms, the CART algorithm had the maximum estimation precision compared to CHAID and Ex-CHAID algorithms. Although the three algorithms could estimate the actual and predicted BW, CART had better goodness of fits, such as Pearson’s correlation coefficient (r), R^2^, and Adj. R^2^ and least RMSE and SD ratio.

**Table 3:** Goodness of fit criteria for CART, CHAID, and Ex-CHAID algorithms.

Statistics	CART	CHAID	Ex-CHAID	Decision
Pearson’s correlation coefficient (r)	0.91	0.80	0.81	Greater is better
Coefficient of determination (R^2^%)	89.23	84.91	83.88	Greater is better
Adjusted coefficient of determination (Adj. R^2^%)	83.33	81.99	81.70	Greater is better
RMSE	11.22	12.69	13.00	Smaller is better
SD ratio	0.32	0.39	0.40	Smaller is better
CV	6.06	5.11	5.56	Smaller is better
MAPE	11.98	13.72	14.38	Smaller is better
RAE	0.25	0.24	0.25	Smaller is better
AIC	260.14	256.11	258.41	Smaller is better

CART=Classification and regression trees, CHAID=Chi-square automatic interaction detection, Ex-CHAID=Exhaustive Chi-square automatic interaction detection, RMSE=Root-mean-square error, SD ratio=Standard deviation ratio, CV=Coefficient of variation, MAPE=Mean absolute percentage error, RAE=Relative approximation error, AIC=Akaike information criterion

## Discussion

In the absence of weighing scales in remote communities, the BW of goats can be estimated with high precision from linear body measurements to assist in marketing, breeding, feeding, and veterinary activities [20]. First, this present research established the association between BW and linear body measurement properties of Kalahari Red goats using Pearson’s correlation. The correlation results demonstrated that BW had an extremely positive, statistically substantial correlation with HG, BL, RH, and WH; nevertheless, it was correlated with HW. For the phenotypic correlation result of bucks, BW had a positive, substantial, and statistical correlation to all linear body measurements. The findings support the study of Iqbal *et al*. [21] in Beetal goat; additionally, they align with the reports of Yilmaz *et al*. [22] in Karya sheep, where it was established that HG functioned as the ideal variable to evaluate BW. The findings from this study demonstrate a positive correlation between linear body measurements and BW. This implies that an increment in linear body measurements will cause BW to increase and vice versa. In bucks, a positive association between BW and linear body measurements suggests that increasing the linear body measurements increases BW. In addition, HG, BL, HW, WH, and RH can be chosen when improving BW and vice versa.

This research aimed to find the ideal statistical algorithm among CART, CHAID, and Ex-CHAID for calculating BW for Kalahari Red goats. Among the three algorithms, CART demonstrated the maximum coefficient of determination and least standard deviation ratio that validate it as the best model for estimating BW. Tyasi *et al*. [23] also established that the CART was an ideal algorithm with an R^2^ of 83% as compared with CHAID (R^2^ = 66%) and Ex-CHAID (R^2^ = 64%). Nevertheless, Celik and Yilmaz [19] compared CART, CHAID, and Ex-CHAID and established that CHAID was the ideal algorithm with an R^2^ of 71.58% and SD ratio of 0.53 for estimating BW in dogs. Abbas *et al*. [10] compared CART, CHAID, and Ex-CHAID for predicting BW for Thalli sheep and indicated that CHAID was the ideal algorithm with the predicted 64.48% R^2^ and 0.59 SD ratio. The CART demonstrated that BL, HG, and wither height play critical roles in BW, inferring it can be chosen to enhance BW since it was established to influence BW strongly. This study also demonstrated that sex played no substantial role in the BW of Kalahari Red goats. Our records disagree with the findings of Mathapo and Tyasi [11] on Boar goats, and the investigation of Eyduran *et al*. [13] on indigenous Beetal Goat of Pakistan revealed that sex played a crucial role in BW. The differences may be due to the application of varying species; however, our findings agree with the reports of Celik [24], which indicated that BL has a substantial role in BW.

## Conclusion

Results have indicated a positive and substantial association between BW and linear body measurement characteristics in Kalahari Red goats, implying that BW can be estimated from linear boy measurement properties. HG, BL, RH, WH, and HW could enhance BW in does, whereas HG, BL, HW, WH, and RH could be chosen for breed enhancement in bucks. The study indicates that the CART is the best statistical algorithm to estimate the BW of Kalahari Red goats. CART reports indicated that BL, HG, and WH, respectively, play a substantial role in the BW of the Kalahari Red goat. Findings from the research will aid breeders and extension officers in advising rural farmers on how to predict the BW of their animals using linear body measurements because of the lack of weighing scales.

## Authors’ Contributions

KM, KMM, TLT, and MCS: Designed the experiment. KMM: Analyzed the data. KM, KMM, and MCS: Performed fieldwork and wrote the manuscript. TLT: Revised, read, and edited. All authors have read and approved the final manuscript.
